# Superhydrophobic Surfaces as a Potential Skin Coating to Prevent Jellyfish Stings: Inhibition and Anti-Tentacle Adhesion in Nematocysts of Jellyfish *Nemopilema nomurai*

**DOI:** 10.3390/ma17235983

**Published:** 2024-12-06

**Authors:** Yichen Xie, Yuanyuan Sun, Rongfeng Li, Song Liu, Ronge Xing, Pengcheng Li, Huahua Yu

**Affiliations:** 1 CAS and Shandong Province Key Laboratory of Experimental Marine Biology, Institute of Oceanology, Chinese Academy of Sciences, Qingdao 266071, China; dhc200812@163.com (Y.X.); sunyuanyuan@qdio.ac.cn (Y.S.); rongfengli@qdio.ac.cn (R.L.); sliu@qdio.ac.cn (S.L.); xingronge@qdio.ac.cn (R.X.);; 2 University of Chinese Academy of Sciences, Beijing 100049, China; 3 Laboratory for Marine Drugs and Bioproducts, Qingdao Marine Science and Technology Center, Qingdao 266237, China

**Keywords:** nano materials, superhydrophobic surface, bioadhesion, nematocysts, jellyfish stings, nemopilema nomurai, nanocellulose, nanochitin

## Abstract

The development of skin-protective materials that prevent the adhesion of cnidarian nematocysts and enhance the mechanical strength of these materials is crucial for addressing the issue of jellyfish stings. This study aimed to construct superhydrophobic nanomaterials capable of creating a surface that inhibits nematocyst adhesion, therefore preventing jellyfish stings. We investigated wettability and nematocyst adhesion on four different surfaces: gelatin, polydimethylsiloxane (PDMS), dodecyl trichlorosilane (DTS)-modified SiO_2_, and perfluorooctane triethoxysilane (PFOTS)-modified TiO_2_. Our findings revealed that an increase in hydrophobicity significantly inhibited nematocyst adhesion. Furthermore, DTS-modified sprayed SiO_2_ and PFOTS-modified sprated TiO_2_ were further enhanced with low-surface-energy substances—cellulose nanofibers (CNF) and chitin nanocrystals (ChNCs)—to improve both hydrophobicity and mechanical strength. After incorporating CNF and ChNCs, the surface of s-TiO_2_-ChNCs exhibited a contact angle of 153.49° even after undergoing abrasion and impact tests, and it maintained its hydrophobic properties with a contact angle of 115.21°. These results indicate that s-TiO_2_-ChNCs can serve as an effective skin coating to resist tentacle friction. In conclusion, this study underscores the importance of utilizing hydrophobic skin materials to inhibit the adhesion of tentacle nematocysts, providing a novel perspective for protection against jellyfish stings.

## 1. Introduction

Jellyfish sting events are considered to be public health and safety issues in various coastal regions worldwide, causing various skin reactions, such as local burning, pruritus, a stinging sensation, linearly arranged erythema, papules, etc. In severe cases, these stings can lead to fatalities [1]. According to the estimation of the National Institute of Health, over 150 million individuals are exposed to the threat of jellyfish stings each year, encompassing fishermen, tourists, military personnel, etc. [2]. Currently, the primary methods for protection against jellyfish stings include the use of anti-shark nets and the wearing of specialized jellyfish suits to keep jellyfish away from human beings [3]. Applying sunscreen has been shown to provide a protective effect against jellyfish stings by preventing the discharge of jellyfish nematocysts [4]; however, research on skin-protective materials against jellyfish stings is limited at present.

The scyphozoan *Nemopilema nomurai* (*N. nomurai*) is one of the most dangerous jellyfish in the Chinese coastal area, where jellyfish blooms occur each year [5]. As a kind of hazardous material, *N. nomurai* venom is responsible for jellyfish stings and is stored in specific subcellular organelle structures called nematocysts. Nematocysts contain signaling proteins that trigger the discharge signal [6] and are structured commonly existing in cnidarians such as jellyfish, sea anemones, coralline, and hydra. The discharge of nematocysts occurs in response to specific external mechanical or chemical stimuli. These structures are propelled at a high velocity to deliver venom subcutaneously, serving purposes of self-defense or predation [6,7]. Regarding the occurrence of jellyfish stings, it is essential to inhibit or intervene in the discharge of nematocysts to prevent venom from entering the human body and subsequently triggering a series of symptoms. The most straightforward and practical approach involves identifying a protective material that serves as a barrier for the skin, therefore minimizing contact between jellyfish tentacles or nematocysts and human skin.

Superhydrophobic surfaces are characterized by special wettability surfaces with a static contact angle greater than 150° and a rolling angle of less than 10°. In recent years, superhydrophobic surfaces with special wettability have sparked a surge of research interest. Due to their unique wetting performance, they possess numerous outstanding properties such as oil–water separations [8], self-cleaning [9], medical materials [10], and corrosion resistance [11], and they have demonstrated significant research and application value in biomedical fields such as in antibacterial coatings [12] and the cryopreservation of living matter [13] in recent years. In addition to these prevalent applications, superhydrophobic surfaces have drawn significant attention regarding the adhesion of cells [14], bacteria [15], and biological macromolecules [16]. For instance, PDMS/TiO_2_ nanocomposite superhydrophobic films demonstrated cell-adhesion-repellent behavior [15], and the quantity of surface-adhered bacteria decreased significantly (>99.8%) on a polyfluoroalkyl-modified TiO_2_ superhydrophobic surface [17], and the value of protein adsorbed on a TiO_2_-PLA-1 membrane significantly decreased in comparison with the original PLA membrane in one study [18]. The adhesion behavior of superhydrophobic surfaces has already become an important research topic for researchers in various fields, being an important step in the interaction process between materials and biological bodies. Therefore, it is reasonable to infer that superhydrophobic surfaces might prevent jellyfish stings by exerting an inhibitory behavior on the adhesion of *N. nomurai* nematocysts. However, the durability of a superhydrophobic surface under different physical stresses remains a significant challenge [19], as it is crucial for practical applications.

Nanocellulose materials are currently extensively applied in functional membranes in industries such as the food industry and biomedical materials [20], showing many advantages. First, nanocellulose materials with a high crystallinity, specific surface area, and mechanical strength are typically utilized as nanofillers and mixed with the functional membrane matrix to enhance mechanical performance [21]. Second, due to their inherent excellent film-forming ability, nanocellulose materials can serve as the matrix materials for functional films [22]. Apart from forming films independently, nanocellulose materials can also be compounded with other materials to form films [23]. Similarly, chitin nanocrystals, as an important natural crystalline polysaccharide, exhibit outstanding hydrophilicity, antibacterial properties, chemical reactivity, biocompatibility, and biodegradability and can be employed in the preparation of functional gels, film-forming materials, wound dressings, etc. [24,25,26]. Nanocellulose and nanochitin can be used as a substrate for superhydrophobic coatings; for instance, modified nano-SiO_2_ was deposited on the surface of a composite film via screen printing, forming a rough nanostructure that resulted in high-strength, transparent, and superhydrophobic nanocellulose/chitin film [27]. To achieve a durable, waterproof, and superhydrophobic surface in underwater environments for jellyfish protection, it is essential to enhance the mechanical strength of the film. Nanocellulose and nanochitin, as biocompatible and degradable materials, demonstrate promising potential for improving the mechanical strength of superhydrophobic materials for jellyfish sting protection.

Hence, it is crucial to investigate skin-protection materials that can effectively prevent the adhesion of nematocysts and improve the mechanical water resistance of such materials to solve the issue of jellyfish stings. In this study, we examined the adhesion behavior of surfaces toward jellyfish nematocysts, including two superhydrophobic surfaces. Furthermore, we modified the structure and mechanical strength of the superhydrophobic surface using nanocellulose and nanochitin and are devoted to finding a high-strength jellyfish superhydrophobic protection coating.

## 2. Materials and Methods

### 2.1. Chemicals and Reagents

Cellulose nanofiber (CNF) and chitin nanocrystals (ChNCs) used in the experiment were prepared by TEMPO oxidation with a solid content of about 1.0 wt.%. Perfluorooctane trichlorosilane (PFTCS), perfluorodecyl trimethoxysilane (PFTTS), perfluorooctane triethoxysilane (PFOTS), perfluorodecyl trimethoxysilane (PFDTS), dodecyl trichlorosilane (DTS), P25-TiO_2_, nano-TiO_2_ (100 nm), nano-SiO_2_ (30 nm) were purchased from Macklin Chemicals (Shanghai, China), PDMS (Dow Corning^®^ Sylgard) was purchased from Dow Corning (Midland, MI, USA), L-PDMS (KF-96A-6T) was purchased from Shin-Etsu Chemical (Tokyo, Japan), 0.5 mm spray pen (15–30 psi). Bama pig skin was purchased from YSKD Bio-technology (Beijing, China). All the other reagents used were of analytical grade.

### 2.2. Nemopilema Nomurai Tentacles Collection

**Figure 1:**
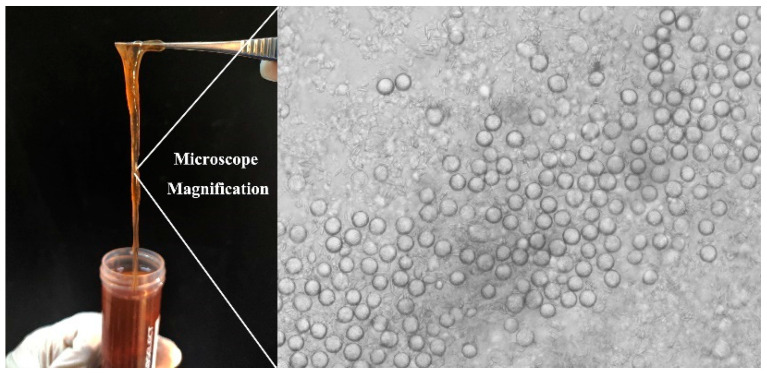
*Nemopilema Nomurai* tentacle sample and nematocysts on surface, bar = 100 μm.

Specimens of the jellyfish *Nemopilema nomurai* (*N. nomurai*) were collected from Laoshan Bay in Qingdao, China, in August 2023. The jellyfish tentacles were excised manually with scissors from the live specimens and were transported to the laboratory under 4 °C conditions and subsequently stored at −80 °C. The nematocyst distribution (Figure 1) on the tentacle surface was observed by an optical microscope at room temperature.

### 2.3. Surface Material Preparation

#### 2.3.1. Gelatin and PDMS Surface Preparation

The gelatin surface was prepared by curing with 10% (*w*/*w*) gelatin solution. PDMS surface was prepared by Sylgard 184 silicone elastomer kit. The prepared suspension was added to the slide and cured at 60 °C for 12 h until the curing surface was formed.

#### 2.3.2. Preparation of Superhydrophobic SiO_2_ and TiO_2_

The preparation process of superhydrophobic SiO_2_ and TiO_2_ suspension was as follows. For superhydrophobic SiO_2_, DTS can react with SiO_2_ efficiently to fabricate superhydrophobic surfaces [28]. A total of 2 g 30 nm SiO_2_ was added to 40 mL cyclohexane into a round-bottom flask, then 1 mL DTS was added, heated, and stirred for 2 h. After the reaction, DTS-modified SiO_2_ suspension was filtered and dried at 353 K for 12 h to obtain DTS-modified SiO_2_ powder. A total of 0.2 g DTS-modified SiO_2_ powder was dispersed in 10 mL ethanol, stirring for 10 min, and ultrasound for 5 min to obtain superhydrophobic SiO_2_. For TiO_2_, PFOTS is commonly used for surface modification [29]. A total of 2 mL PFOTS was added into 50 mL ethanol and stirred for 1.5 h at 60 °C, followed by adding 3 g P25-TiO_2_ and 3 g TiO_2_ (100 nm) slowly. PFOTS-modified TiO_2_ suspension was prepared after stirring at 600 rpm for 4 h at room temperature. The spraying of superhydrophobic SiO_2_ and TiO_2_ was conducted at a medium speed (10–15 cm) with an air pump spray pen (15 psi), and the material surface was fabricated following complete evaporation of ethanol for 30 s.

#### 2.3.3. Preparation of CNF and ChNCs Composite Materials

First, 1.0 wt.% CNF ethanol suspension was mixed with PDMS, L-PDMS, and squalane at a ratio of 1:20 (*w*/*w*). Second, 1.0 wt.% CNF ethanol suspension was mixed with PFOTS, PFTCS, PFTTS, PFDTS, and DTS. The volume ratio of silane and CNF suspension was 1:50. A total of 20 mL CNF suspension was modified by adding 400 μL silane, and the suspension was stirred at 600 rpm at room temperature for 5 h. Finally, for the mixture of superhydrophobic suspension with CNF and ChNCs, 1.0 wt.% CNF was mixed with DTS-modified SiO_2_ and PFOTS-modified TiO_2_ suspension (as described in 2.3.2) at ratios of 1:1, 10:1, 10^2^:1, and 10^3^:1, respectively. ChNC suspension was prepared in the same way as CNF, and the Bama pig-skin surface material spraying process was the same.

#### 2.3.4. Mechanical Strength Test of Surfaces

**Figure 2:**
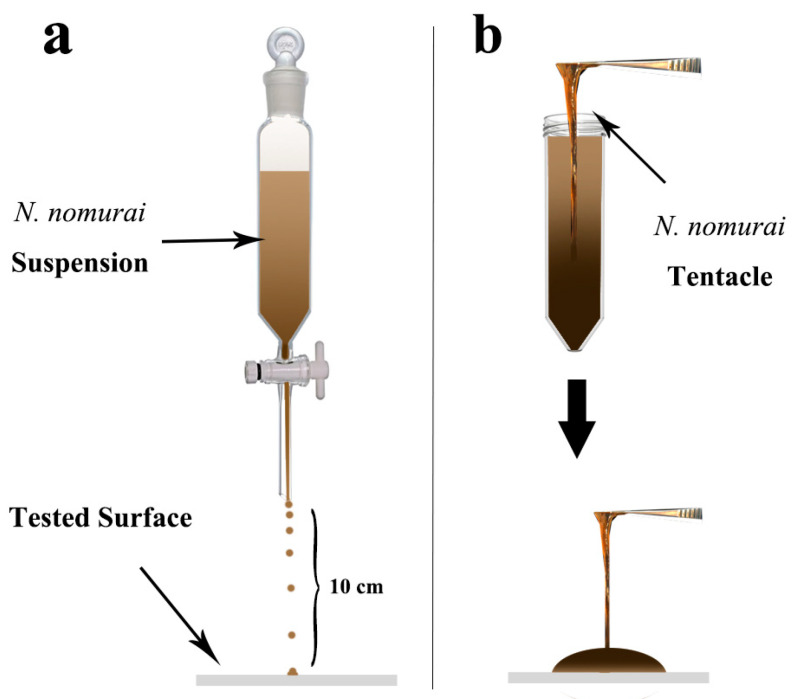
Abrasion and impact test operation mode. (**a**) Jellyfish suspension impact test, (**b**) Jellyfish tentacle impact test.

(1). Abrasion test. For the experiment of adhesion by tentacles, intact *N. nomurai* tentacles were selected for the experiment, and 1 mL of tentacle suspension was carefully applied to the material’s surface (shown in Figure 2b). After a 30 s adhesion, the surface was rinsed 3 times with PBS buffer (pH = 7.4, 10 mM). (2). Impact test. For the experiment of jellyfish suspension (the suspension after jellyfish autolysis) impact resistance test, the supernatant containing jellyfish toxin and protein mixture was used, and the supernatant was obtained by centrifuging the tentacle suspension at 1000× *g* for 5 min. The jellyfish suspension impacts the test surface at a distance of 10 cm above with the same flow rate using a barrel-shaped separatory funnel (shown in Figure 2a). The impact time ranged from 15 s to 90 s. After the jellyfish suspension impact was completed, the liquid on the surface of the material was carefully removed with filter paper. (3). Seawater immersion test. The superhydrophobic material was sprayed on Bama pig skin and subsequently immersed in 5 L natural seawater in a square vessel, shaken at 200 rpm for 2 h. The surface contact angle was measured.

### 2.4. Contact-Angle Measurement

After modification and tentacle adhesion, a video-based contact-angle measurement system (JC2000D1) was employed to determine the water contact angle (CA) values of the samples. The CA measurements of each sample were conducted at least three times across the sample surfaces using the sessile drop method by dispensing 5 μL drops of ddH_2_O on the surface samples. Sliding-angle (SA) and contact-angle hysteresis were measured by Scientific LSA100S-T (LAUDA, Lauda-Königshofen, German). All values were measured under the ambient laboratory conditions at a temperature of 25 °C, in parallel, three times in different areas of the surfaces.

### 2.5. Scanning Electron Microscope Characterization

Morphologies of the adhesion of nematocysts and surface morphology on gelatin, PDMS, and the superhydrophobic surfaces were observed by a digital scanning electron microscope. After treatment, the surface was fixed with 2.5% glutaraldehyde in PBS (pH 7.4) for 2 h and dehydrated in a series of increased concentrations of ethanol (30, 50, 70, 80, 90, and 100%) for 10 min at 4 °C. The cells were dried by a critical point dryer (Hitachi-HCP, Hitachi, Tokyo, Japan), sputter-coated with platinum (MC1000, Hitachi, Japan), and examined with a scanning electron microscope (S-3400N, Hitachi, Japan) operated at 5 kV.

### 2.6. X-Ray Photoelectron Spectroscopy Characterization

The X-ray photoelectron spectroscopy (XPS) analysis was conducted via a Thermo Scientific K-Alpha (Waltham, MA, USA). The pressure in the sample chamber is less than 2.0 × 10^−7^ mbar, the spot size is 400 μm, and the working voltage is obtained at 12 kV with filament current 6 mA.

### 2.7. Statistical Analysis

Multiple samples were tested, and the results were reported as average values ± standard deviation.

## 3. Results and Discussion

### 3.1. Superhydrophobic Surface Tentacles Adhesion and Characterization

**Figure 3:**
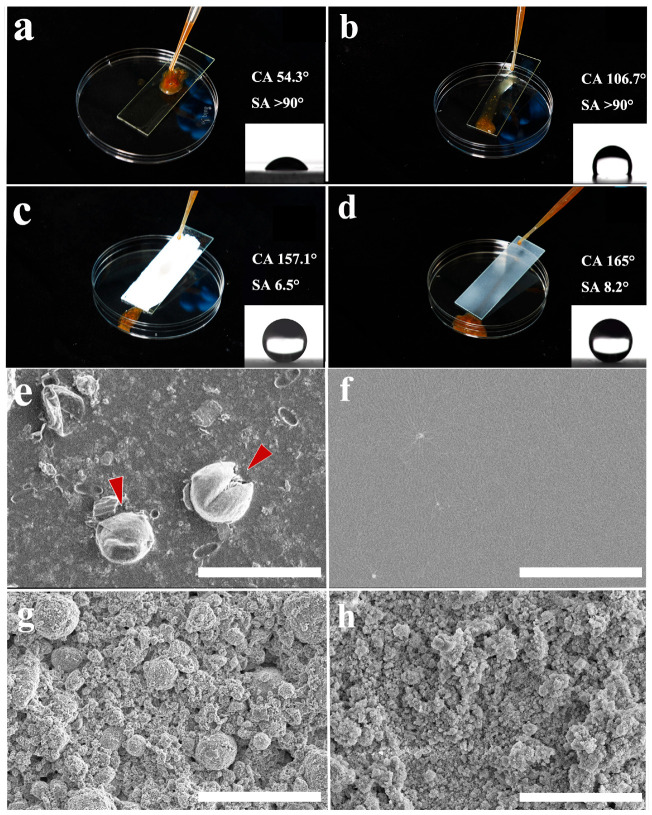
Images of the adhesion behavior of surfaces and morphology of the surface after jellyfish adhesion. (**a**) Gelatin surface, (**b**) PDMS surface, (**c**) PFOTS-modified sprayed TiO_2_, (**d**) DTS-modified sprayed SiO_2_, (**e**) Gelatin surface, the red arrows represent the cracked undischarged nematocysts, (**f**) PDMS surface, (**g**) PFOTS-modified sprayed TiO_2_, (**h**) DTS-modified sprayed SiO_2_, bar = 50 μm.

The wettability of gelatin, PDMS, DTS-modified sprayed SiO_2_, and PFOTS-modified sprayed TiO_2_ was investigated through water contact angle (CA) and slide angle (SA) measurements. The CA significantly increased from 54.3° for gelatin to 165° for DTS-modified sprayed SiO_2_ (as shown in Figure 2). When suspended droplets containing tentacle fragments of *N. nomurai* were added to inclined surfaces, it was observed that the nematocyst suspension on the gelatin surface (CA = 54.3 ± 5°, SA > 90°) exhibited minimal sliding ability, while tentacular fragments floated on the surface of PDMS (CA = 106.7 ± 2°, SA > 90°) and slid at a slower speed with tissue fluid traces left behind during the process (Figure 3a,b). In experiments using two superhydrophobic surfaces, DTS-modified sprayed SiO_2_ and PFOTS-modified sprayed TiO_2_, the results revealed that jellyfish suspension formed a ball-shaped structure on the surface and rapidly rolled down to the bottom. In terms of transparency, TiO_2_ appeared entirely opaque in white color, whereas SiO_2_ was semi-translucent (as shown in Figure 3c,d). The transition from hydrophilicity to hydrophobicity could significantly inhibit the adhesion of jellyfish nematocysts, suggesting a potential association between low surface energy and inhibition of jellyfish nematocyst adhesion.

The surface of jellyfish tentacles contains a considerable number of nematocysts that cause jellyfish stings. Our results indicate that the water-repellent hydrophobic material PDMS did not adhere to nematocysts (Figure 3f). We hypothesize that the adhesion of nematocysts would be greatly reduced with an improvement in surface-wetting properties, and a certain level of hydrophobicity may effectively inhibit the adhesion of jellyfish nematocysts.

Regulating the nematocysts’ adhesion on the skin surface is a key strategy in the field of jellyfish sting protection research. In this study, the adhesion behavior of several hydrophobic, superhydrophobic surfaces, and modified superhydrophobic surfaces were investigated. Scanning electron microscopy (SEM) analysis was employed since this approach can distinguish the adhesion behavior differences of individual nematocysts intuitively, and the characterization results are also shown in Figure 3. The surface morphology of gelatin and PDMS film were smooth, and nematocysts of *N. nomurai* on the gelatin surface were found (red arrows in Figure 3e), while no nematocysts were found on the PDMS surface (Figure 3f). Most nematocysts on the gelatin surface remained undischarged, with some displaying intact morphology and others exhibiting cracks that exposed the internal spiral structure tubules and inner part of the capsule wall. No evidence of nematocyst adhesion was observed on DTS-modified sprayed SiO_2_ and PFOTS-modified sprayed TiO_2_. As depicted in Figure 3c,d, the two superhydrophobic surfaces were markedly non-smooth and displayed extreme roughness in sharp contrast to gelatin and PDMS. The superhydrophobic PFOTS-modified sprayed TiO_2_ (s-TiO_2_) surface featured grain sizes ranging from 2 μm to 20 μm, while the grain sizes of superhydrophobic DTS-modified sprayed SiO_2_ (s-SiO_2_) were less than 10 μm. In summary, it was evident that the superhydrophobic surface exhibits non-adhesive characteristics towards *N. nomurai* nematocysts upon transient contact; however, long-lasting abrasion may lead to damage to the superhydrophobic structure.

### 3.2. Impact Test of Jellyfish Suspension on the Composite Material of CNF and ChNCs

**Figure 4:**
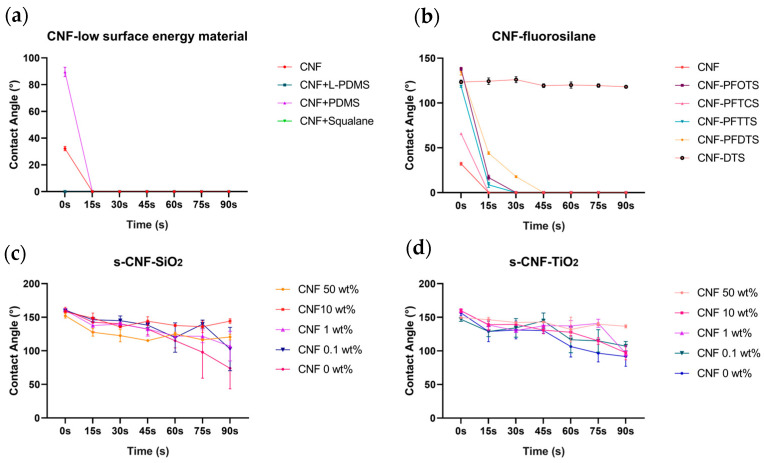
The variations in the CA of different CNFs after being impacted by *N. nomurai* suspension for different durations. (**a**) Post-impact CA of CNF-low-surface-energy material, (**b**) CNF-fluorosilane, (**c**) s-SiO_2_-CNF, (**d**) s-TiO_2_-CNF.

Nanocellulose materials (CNF), including nanocellulose derivatives modified by chemical methods, are among the most promising green and renewable materials that have emerged along with the development of nanotechnology. Depending on requirements, nanocellulose materials with different surface modifications can be employed to enhance the bonding strength with the matrix, therefore facilitating the functional membranes in achieving the desired mechanical properties. We conducted experiments to investigate the potential for modifying CNF with composite materials containing hydrophobic substances. Several low-surface-energy substances (L-PDMS, PDMS, and squalane) were first tested. The CA of CNF was 32.20°, exhibiting hydrophilic properties. The result showed that four surfaces (CNF, CNF + L-PDMS, CNF + PDMS, CNF + squalane) exhibited more hydrophilicity character after being impacted with jellyfish suspension (shown in Figure 4a), indicating that CNF was difficult to compound with low-surface-energy materials. Due to the presence of hydroxyl groups on the surface of cellulose nanofibrils (CNF), we conducted experiments to investigate the hydrophobic modification effect of various fluorosilanes commonly used in modifying nano TiO_2_ and SiO_2_. After CNF reacted with fluorosilanes, the hydrophobicity was enhanced, and the hydrophilicity of CNF was transformed to hydrophobicity character in most cases (Figure 4b, CNF-PFOTS 138.08°, CNF-PFTCS 65.78°, CNF-PFTTS 118.53°, CNF-PFDTS 134.84°, CNF-DTS 123.48°). After being impacted with the jellyfish suspension for 90 s, only the DTS-modified CNF surface remained highly hydrophobic, with a CA of 118.01°. We believe that DTS might react with hydroxyl on the structure of CNF, thus achieving a change in hydrophobicity. Finally, experiments of mixing superhydrophobic suspension directly with CNF were carried out. The CNF was combined with superhydrophobic materials, including s-TiO_2_ and s-SiO_2_, at varying ratios. The results demonstrated that the mechanical strength against jellyfish suspension of superhydrophobic materials was significantly enhanced. Furthermore, all surfaces treated with s-SiO_2_ achieved a superhydrophobic level without contacting jellyfish suspension (CNF 50 wt.% CA = 152.19°, CNF 10 wt.% CA = 158.03°, CNF 1.0 wt. % CA = 160.44°, CNF 0.1 wt.% CA = 160.33°, n = 3) and part of the surfaces with s-TiO_2_ reached superhydrophobic level (CNF 10 wt. % CA= 159.67°, CNF 1.0 wt.% CA = 159.70°, n = 3). Detailed data are shown in Supplementary Materials Table S1. With the increase of the impact time from 15 s to 90 s, the CA on the surface slightly reduces (Figure 3c, d). The CA of 10 wt.% s-SiO_2_-CNF and 50 wt.% s-TiO_2_-CNF maintained an exceptionally high level of hydrophobic performance even after a 90 s impact, thus leading to the selection of these compositions for the skin tentacle protection test. In conclusion, CNF enhanced the anti-impact properties of superhydrophobic materials against jellyfish suspension.

**Figure 5:**
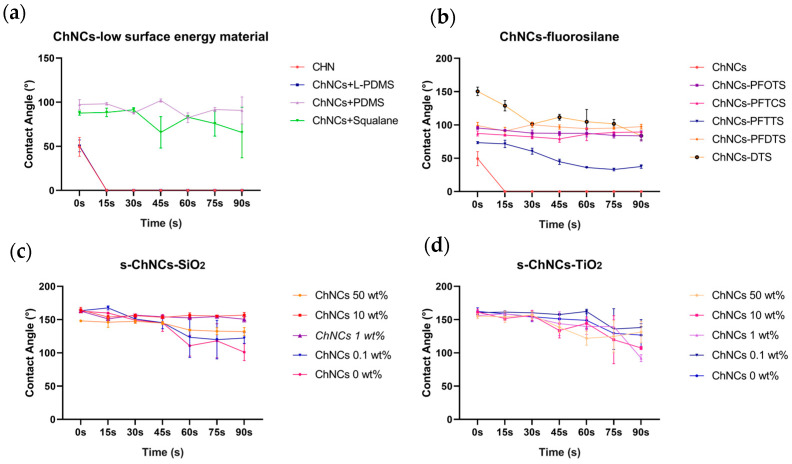
The variations in the CA of different ChNCs after being impacted by *N. nomurai* suspension for different durations. (**a**) ChNCs-low-surface-energy material compound post-impact CA, (**b**) ChNCs-fluorosilane, (**c**) s-SiO_2_-ChNCs, (**d**) s-TiO_2_-ChNCs.

Nanochitin (ChNCs) is similar to nanocellulose and has numerous applications in green biofilm materials as well as in strengthening the mechanical properties of materials, therefore, we performed a similar experiment for ChNCs as for CNF (the results were shown in Figure 5). The CA of ChNCs was 49.40° which was higher than CNF. After ChNCs was mixed with low-surface-energy material, the CA after 90 s jellyfish suspension impact was 90.64° for the PDMS mixture and 65.64° for the squalane mixture. The results suggested that ChNCs had superior compatibility with low-surface-energy substances compared to CNFs. After the mixture and reaction of ChNCs with 5 fluorosilanes, the CA was higher than that without modification (ChNCs-PFOTS 95.49°, ChNCs-PFTCS 87.21°, ChNCs-PFTTS 73.37°, ChNCs-PFDTS 99.00°, ChNCs-DTS 150.47°), and CA slightly changed with longer jellyfish suspension contact. In comparison with the CNF group, the fluorosilane-modified ChNCs demonstrated better material strength to the jellyfish suspension. In the fluorosilanes group, DTS-modified ChNCs exhibited superhydrophobic character before impact, which indicated that the hydroxyl of the ChNCs structure had more reactions with DTS compared with CNF. The s-TiO_2_ and s-SiO_2_ were mixed with ChNCs with different ratios, and the mechanical strength of superhydrophobic materials was strengthened like the CNF group, slightly different from the CNF group, part of the surfaces with s-SiO_2_ attained superhydrophobic level without jellyfish suspension contact (ChNCs 10 wt.% CA = 164.47°, ChNCs 1.0 wt. % CA = 162.67°, ChNCs 0.1 wt.% CA = 163.35°, n = 3) and all the surfaces with s-TiO_2_ attained superhydrophobic level (ChNCs 50 wt.% CA = 155.08°, ChNCs 10 wt.% CA = 161.22°, ChNCs 1.0 wt.% CA = 156.12°, ChNCs 0.1 wt.% CA = 160.60°, n = 3). Detailed data can be found in Supplementary Materials Table S2. Similar to CNF, 10 wt.% s-SiO_2_-ChNCs and 0.1 wt.% s-TiO_2_-ChNCs were selected for the skin tentacle protection test.

Our experiments revealed that superhydrophobic surfaces have excellent adhesion inhibition behavior for nematocysts, as shown in Figure 3c, d; however, due to the extreme fragility of superhydrophobic surfaces, it is rather challenging for them to exert their functions in practical situations. Nanocellulose and nanochitin have been successfully used for the preparation of superhydrophobic suspensions as structural reinforcing material. A superhydrophobic surface with a CA of 150.2° was fabricated by blending HDTMS-modified nano-SiO_2_ and nanocellulose [30]. The superhydrophobic nanocellulose membranes were prepared by directly using bamboo fiber powder, 5% PVA, hydrochloric acid dopamine, and hexadecyltrichlorosilane as the surface modifier [31]. We used nanocellulose and nanochitin to enhance the structural properties of two superhydrophobic surfaces (s-SiO_2_ and s-TiO_2_) in this study, and the results showed that the impact resistance of the two superhydrophobic surfaces was improved with the addition of CNF and ChNCs, the optimal mixing ratio of material strength for s-SiO_2_ and s-TiO_2_ varies, for s-SiO_2_, the optimal concentration of CNF and ChNCs were both 10 wt.%, respectively, for s-TiO_2_, the optimal concentration of CNF and ChNCs were 50 wt.% and 0.1 wt.%, respectively. It is commonly believed that using nanocellulose alone is not sufficient to create a superhydrophobic surface, and the key to forming a superhydrophobic surface lies in fluorinated-modified nanoscale silica, while the presence of nanocellulose and chitin serves to enhance mechanical strength.

### 3.3. Anti-Tentacle Abrasion of Superhydrophobic Composite Materials on Skin Surface

Gelatin is frequently employed as a substitution for the skin surface in certain studies; however, there exists a considerable disparity in surface topography and chemical composition between gelatin and human skin. Since the amino acid composition of type I collagen in the skin of Bama pigs demonstrates physicochemical characteristics similar to those of human skin, including surface morphological characteristics [32], Chinese Bama pig skin was used to simulate the contact between the surface of human skin and the tentacles of *N. nomurai*. We selected four superhydrophobic surface materials (as shown in Table 1) with the most effective anti-jellyfish suspension behavior and applied them to the surface of pig skin for adhesion testing with jellyfish tentacles. The superhydrophobic surface can be successfully sprayed onto the pig-skin surface environment. However, the results revealed that most of the superhydrophobic surfaces constructed on the skin were relatively delicate, and the destructive effects of jellyfish tentacles on these surfaces were significantly destroyed after contact. Among those materials, s-TiO_2_-ChNCs (0.1 wt.%) still maintained a relatively large contact angle (CA = 115.21°) after being impacted by the tentacles. The duration of contact with a jellyfish’s tentacles is typically brief, lasting only a few seconds, and the examination of tentacles for extended periods of contact represents an extreme scenario. Therefore, the tentacle abrasion of superhydrophobic structure in actual condition may not be so severe. Even more noteworthy is the fact that the superhydrophobic skin coating must effectively resist saturation by seawater for a long time, which is a more practical issue.

**Figure 6:**
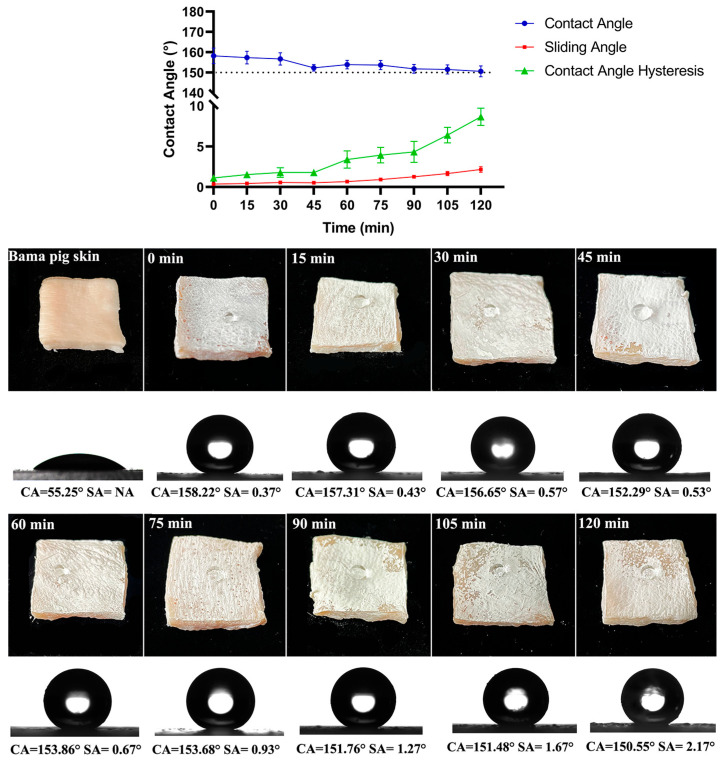
Seawater immersion test for s-TiO_2_-ChNCs on Bama pig skin, contact angle (CA), sliding angle (SA), contact-angle hysteresis, and images of s-TiO_2_-ChNCs material on the skin surface after seawater immersion from 0 min to 120 min. The NA in the figure indicates that the SA cannot be measured.

The s-TiO_2_-ChNCs (0.1 wt.%), which showed the best properties above, were further tested in seawater, and the result revealed that a 2 h exposure to seawater did not result in obvious erosion or damage to the superhydrophobic properties of s-TiO_2_-ChNCs surface, the CA decreased from 158.22° to 150.55° (as shown in Figure 6). The determination of the Cassie–Baxter state also requires two more data—a sliding angle of less than 10° and CA hysteresis of less than 5°. The results indicate that the sliding angle increased from 0.37° to 2.17° and the CA hysteresis increased from 1.13° to 8.67° with the immersion of seawater, and the detailed data are shown in Supplementary Materials Table S3. After 90 min of immersion in seawater, the CA hysteresis exceeded 5°, while the change of SA did not exceed 1°. Overall, immersion in seawater for less than 90 min will not make the s-TiO_2_-ChNCs on the skin surface lose their superhydrophobic characteristics. In addition to seawater immersion, factors such as ultraviolet (UV) irradiation and temperature can also influence the surface properties. Future studies should investigate these and other real-world factors to better understand their effects on the material.

**Figure 7:**
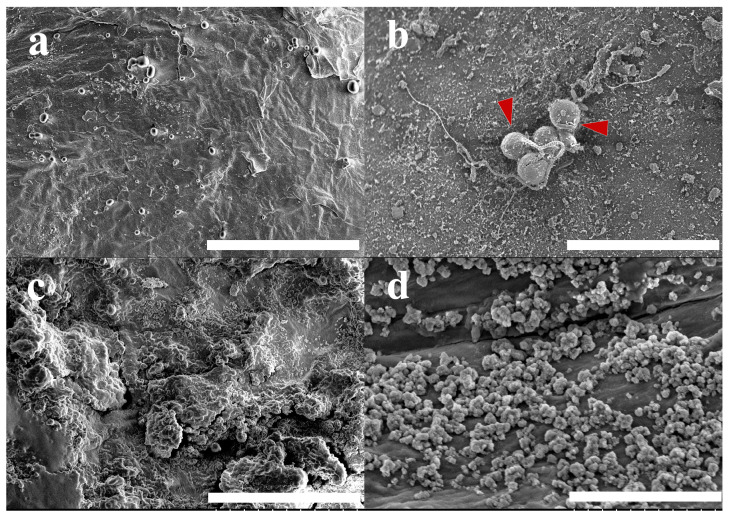
SEM images of jellyfish tentacle contact on Bama pig skin and s-TiO_2_-ChNCs covered skin. (**a**) Bama pig skin before tentacle contact, (**b**) Bama pig skin after tentacle contact, red arrows represent the undischarged nematocysts and tubules, (**c**) s-TiO_2_-ChNCs covered skin before tentacle contact, (**d**) s-TiO_2_-ChNCs covered skin after tentacle contact, bar = 100 μm.

Morphological observations of Bama pig skin and s-TiO_2_-ChNCs-covered skin before and after tentacle contact were also conducted with SEM (Figure 7). Before tentacle contact, the skin surface was relatively smooth and had a small number of impurities. After tentacle contact, undischarged nematocysts could be observed on Bama pig skin, and nematocyst morphology was similar to that of the gelatin surface, with individual nematocysts cracked and exposing the internal tubules (red arrows in Figure 7b). However, for the s-TiO_2_-ChNCs compound, the surface morphology was significantly different from s-TiO_2_ in Figure 3g. Before tentacle contact, the surface exhibited a relatively high roughness, and distinct spherical structures no longer existed (as shown in Figure 7c). This morphology indicated that the nanostructure of ChNCs was coating the surface of the TiO_2_ nanoscale superhydrophobic structure, acting like a binding agent. After tentacle contact, the superhydrophobic structure was destroyed, and some areas were exposed on the skin surface (as shown in Figure 7d), yet the surface still retained a rather rough morphology, and no nematocysts were found on the s-TiO_2_-ChNCs surface, although the superhydrophobic structure was destroyed. We contend that a lower surface energy or a relatively higher CA (for example, PDMS surfaces) can effectively inhibit the adhesion of jellyfish nematocysts, and surface roughness may achieve inhibition of adhesion by reducing the contact area between jellyfish nematocysts and materials.

The morphology of the s-TiO_2_-ChNCs superhydrophobic surface exhibited significant alterations after being abraded by jellyfish tentacles (Figure 7c,d). Additionally, the other three types of surfaces completely lost their hydrophobicity property after tentacle adhesion, resulting in a decrease in CA down to 0° (Table 1). This result indicated that proteins denatured at the superhydrophobic surface, leading to subsequent surface wetting due to the formation of a more hydrophilic interface [18]. Therefore, except for considering the basic physical forces of mucus, the proteins of jellyfish might potentially cause damage to superhydrophobic surfaces. It has been demonstrated that the superhydrophobic surface can capture different kinds of glycoproteins, and the capture of glycoproteins is markedly higher than that of non-glycoproteins [33]. Furthermore, because of the intense hydrophobic–hydrophobic interactions between the hydrophobic functional groups of proteins and the hydrophobic substrates, hydrophobic surfaces can adsorb more proteins than hydrophilic surfaces [34]. The jellyfish mucus on the tentacle’s surface is mainly composed of protein, such as glycoproteins [35], jellyfish toxins [36], as well as enzymatic substances, such as lysozyme [37]. The complex jellyfish protein system is inevitably detrimental to the structure and chemical composition of superhydrophobic surfaces.

**Figure 8:**
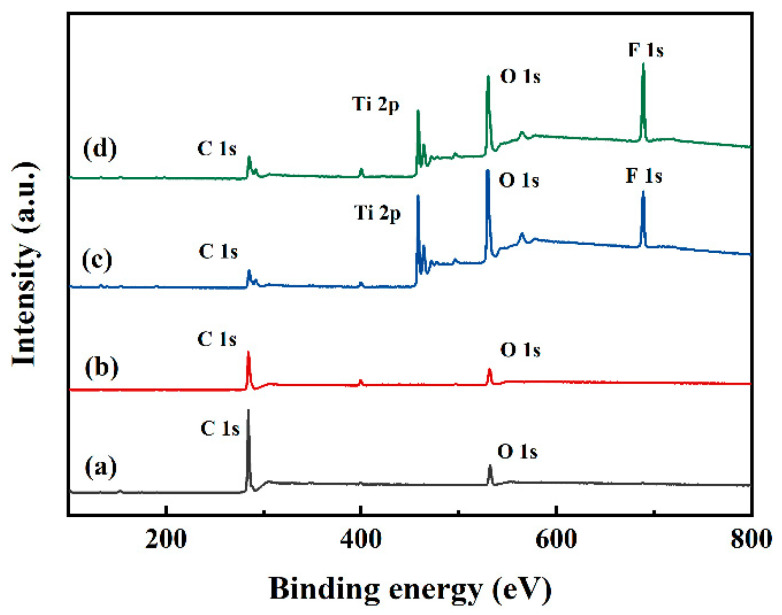
XPS wide-scan spectra for (a) skin, (b) skin-tentacles, (c) s-TiO_2_-ChNCs, and (d) s-TiO_2_-ChNCs-tentacles.

According to the morphology results, the residual amounts of fluorosilane and TiO_2_ on the surface require further characterization; therefore, X-ray photoelectron spectroscopy (XPS) was employed to determine the surface composition of the samples. The wide-scan XPS spectra for the samples, loaded with different contents of nanoparticles, are shown in Figure 8, and the atomic concentrations are listed in Table 2. In the wide-scan spectrum of skin, distinguished O 1s and C 1s peaks could be observed, and S 1s peaks originating from collagen could not be directly observed in the spectrum, indicating that the surface was comprised of organic organisms. Following contact with jellyfish tentacles, the content of C element declined, whereas the contents of O and S elements (Table 2) rose marginally, indicating that tentacles and jellyfish fragments of *N. nomurai* remained on the skin. The cyst wall of the nematocyst contains a relatively large amount of disulfide bonds and collagen [38]; therefore, the increase of the S element may lie in the adhesion of the nematocysts. After being covered with s-TiO_2_-ChNCs, obvious Ti 2p and F 1s peaks were detected on the surface, and the marked reduction in the content of C suggested that the skin was covered by the s-TiO_2_-ChNCs material. Due to the accumulation of jellyfish proteins like glycoproteins, the concentration of C element increased. The surface of nano TiO_2_ was modified with fluoroalkyl silane; however, the peaks of Si 2s and Si 2p were harder to observe in the spectrum. After contact with the tentacles, there was a reduction in the percentages of Ti and O elements (15.58% and 21.63% loss, respectively), indicating the quantity lost due to abrasion of s-TiO_2_-ChNCs by the tentacles. The content of the F element increased after contact with *N. nomurai* tentacles, and we hold the opinion that ChNCs structure covering the fluorosilane chemical bonding to TiO_2_. After the ChNCs layer was destroyed by tentacle abrasion, C-F bonds (on TiO_2_) were exposed. At present, the quantitative research method for the surface adhesion of nematocysts is blank. The differences in surface and interface elements can only indirectly reflect the adhesion behavior of nematocysts; therefore, it is necessary to establish a model for the adhesion of nematocysts.

**Figure 9:**
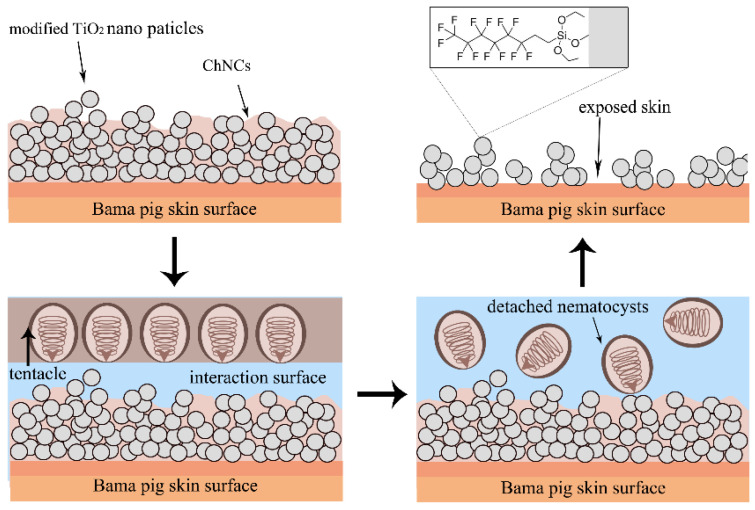
Diagram of the surface contact of the tentacles and s-TiO_2_-ChNCs.

As shown in Figure 9, s-TiO_2_ forms a superhydrophobic layer on the skin surface with the evaporation of ethanol solvent, and ChNCs fill among the nanoparticles, playing a role in strengthening the mechanical strength of the superhydrophobic surface. Upon contact with the s-TiO_2_-ChNCs surface, the tentacles are unable to directly interact with the skin due to the hydrophobic force of the superhydrophobic surface. However, it is important to note that the presence of mucus protein and other complex jellyfish proteins in the seawater environment may potentially compromise the integrity of the superhydrophobic structure. Additionally, the repulsion of isolated nematocysts by the hydrophobic fluorosilane on s-TiO_2_-ChNCs surface is attributed to their composition of hydrophilic collagen protein [38], leading to their removal along with seawater due to hydrophobic force. The disruption of this superhydrophobic surface structure by tentacles, in the absence of any remaining nematocysts, can be ascribed not only to physical forces exerted by tentacles but also significantly influenced by jellyfish proteins.

Superhydrophobic materials have many applications in the field of biomaterials, such as dental implants, sutures, and many medical devices for biometric identification technology. Biocompatibility is a frequently discussed subject [10]. According to the previous study, bone marrow stem cells can retain intact cellular morphology on the fluorosilane-modified silica superhydrophobic surface [39], and superhemophobic surface offers significantly improved (>40%) cell viability as compared to glass [40], demonstrating that many superhydrophobic materials have low cytotoxicity. The s-TiO_2_-ChNCs are coated on the skin surface, and the major chemical substances are TiO_2_ and ChNCs, which possess minimal cytotoxicity [41,42]; therefore, s-TiO_2_-ChNCs may have negligible toxicity to the skin.

## 4. Conclusions

The venom of jellyfish, stored in a specialized subcellular organelle called nematocysts, is the primary cause of jellyfish stings. It is crucial to address the interaction between nematocysts and human skin. Regulating the adhesion of nematocysts on the skin surface presents a potentially effective approach for preventing jellyfish stings; however, research on protective materials for jellyfish stings is currently lacking. Our study revealed the excellent inhibitory behavior of superhydrophobic surfaces against *N. nomurai* nematocyst adhesion. By combining two nanomaterials (CNF and ChNCs) with enhanced mechanical strength to modify the surface, we identified a skin coating named s-TiO_2_-ChNCs that exhibited inhibitory effects against nematocysts adhesion, demonstrating favorable performance in practical tests. The inhibition of nematocyst adhesion was confirmed through SEM and XPS characterization, and s-TiO_2_-ChNCs coating can retain its superhydrophobic characteristics for a long time in the seawater environment. Our findings highlight the efficacy of superhydrophobic surfaces in mitigating interactions between biological adhesives found on nematocysts and diverse substrates, therefore reducing the potential for jellyfish envenomation. This material is recommended for application as a spray onto the skin surface and can be easily washed off with surfactant. Compared to traditional jellyfish suits, it is more cost-effective and accessible, with potential for mass production in the future, and to guarantee the safety of the material, cytotoxicity and related research needs to be investigated in the future.

## Figures and Tables

**Table 1:** The contact angle (CA) before and after the contact of the surface material of the *N. nomurai* tentacles.

Samples	Bama Pig Skin	s-SiO_2_-ChNCs (10 wt.%)	s-TiO_2_-ChNCs (0.1 wt.%)	s-SiO_2_-CNF (10 wt.%)	s-TiO_2_-CNF (50 wt.%)
Before-adhesion	55.25°	141.36°	153.49°	116.43°	144.10°
Post-adhesion	0°	0°	115.21°	0°	0°

**Table 2:** The atomic concentrations (percentage) of different elements on the surface of the skin and TiO_2_-ChNCs material and its corresponding nanocomposites.

Samples	[C]	[O]	[S]	[F]	[Ti]	[Si]
skin	87.21	10.03	0.21	0	0	0
skin-tentacles	85.37	12.69	0.31	0	0	0
s-TiO_2_-ChNCs	27.42	42.76	0.53	13.31	14.47	1.78
s-TiO_2_-ChNCs-tentacles	32.88	36.10	0.63	17.27	11.34	1.50

## Data Availability

The original contributions presented in this study are included in the article. Further inquiries can be directed to the corresponding author.

## Supplementary Materials

The following supporting information can be downloaded at: https://www.mdpi.com/article/10.3390/ma17235983/s1, Table S1: The contact angle (°) after the jellyfish suspension impact test of the CNF surface material, impact time range from 15 s to 90 s (n = 3).; Table S2: The contact angle (°) after the jellyfish suspension impact test of the ChNCs surface material, impact time range from 15 s to 90 s (n = 3); Table S3: The contact angle (°) sliding angle (°) and contact angle hysteresis (°) after sea water immersion, immersion time range from 15 min to 120 min (n = 3).
