# Supplementary material for: Superhydrophobic Surfaces as a Potential Skin Coating to Prevent Jellyfish Stings: Inhibition and Anti-Tentacle Adhesion in Nematocysts of Jellyfish Nemopilema nomurai
Source: Materials (Basel). 2024 Dec 6;17(23):5983. doi: 10.3390/ma17235983 (PMC11643888; doi:10.3390/ma17235983)
Supplement: Supplementary file 1 [file materials-17-05983-s001.zip › materials-3316592-supplementary.pdf]

Supplementary material

Table S1. The contact angle (°) after the jellyfish suspension impact test of the CNF surface material, impact time range from 15 s to 90 s (n=3).

Samples \ CA(°) \ Time	0 s	15 s	30 s	45 s	60 s	75 s	90 s
CNF	32.20±1.61	0	0	0	0	0	0
CNF+L-PDMS	0	0	0	0	0	0	0
CNF+PDMS	89.56±3.50	0	0	0	0	0	0
CNF +Squalane	0	0	0	0	0	0	0
CNF-PFOTS	138.08±1.75	16.82	0	0	0	0	0
CNF-PFTCS	65.78±1.22	0	0	0	0	0	0
CNF-PFTTS	118.53±1.03	8.71±2.74	0	0	0	0	0
CNF-PFDTS	134.84±3.69	44.07±1.46	17.78±1.08	0	0	0	0
CNF-DTS	123.48±2.03	124.31±3.58	126.01±3.35	119.36±2.13	120.00±3.43	119.45±1.96	118.01±1.31
s-SiO_2-CNF							
CNF 50 wt. %	152.19±3.50	127.60±5.66	122.63±9.14	115.33±1.28	125.03±2.74	116.49±1.24	120.54±4.28
CNF 10 wt. %	158.03±6.78	148.78±7.47	135.61±1.15	144.16±6.55	137.70±1.50	135.91±8.45	144.20±3.51
CNF 1.0 wt. %	160.44±2.42	137.62±9.24	139.91±2.79	132.39±10.43	122.10±2.83	121.60±9.54	106.94±22.10
CNF 0.1 wt. %	160.33±0.94	146.32±3.19	144.87±7.13	138.23±3.65	119.79±21.79	139.69±6.02	102.67±32.27
CNF 0 wt. %	161.24±1.56	142.57±5.60	140.21±1.58	132.28±8.31	115.05±10.66	97.91±38.69	73.87±30.41
s-TiO_2-CNF							
CNF 50 wt. %	149.51±0.59	146.44±3.42	142.26±1.35	142.90±1.20	131.85±18.41	139.81±2.39	136.51±2.23
CNF 10 wt. %	159.67±3.05	139.06±7.60	139.45±1.63	130.87±5.48	127.79±4.81	114.84±4.97	97.18±10.47
CNF 1.0 wt. %	159.70±2.44	138.51±8.55	130.10±2.08	137.41±0.78	137.27±7.77	140.98±6.08	96.17±3.58
CNF 0.1 wt. %	146.88±2.90	128.85±7.65	134.24±13.77	144.70±11.76	116.58±19.33	114.85±16.66	107.05±6.90
CNF 0 wt. %	156.47±4.02	128.98±15.19	131.15±13.23	130.33±3.90	106.39±15.50	96.66±13.13	91.56±14.54

Table S2. The contact angle (°) after the jellyfish suspension impact test of the ChNCs surface material, impact time range from 15 s to 90 s (n=3).

Samples \ CA(°) \ Time	0 s	15 s	30 s	45 s	60 s	75 s	90 s
ChNCs	49.40±10.65	0	0	0	0	0	0
ChNCs +L-PDMS	54.49±6.74	0	0	0	0	0	0
ChNCs +PDMS	97.44±5.46	98.23±1.50	87.79±1.33	101.88±1.83	82.39±5.51	91.63±2.27	90.64±15.35
ChNCs +Squalane	87.69±2.42	88.41±4.76	91.46±2.34	65.87±17.84	83.12±1.97	75.77±14.26	65.64±28.64
ChNCs -PFOTS	95.49±2.23	91.73±1.32	87.70±4.60	87.49±2.95	87.39±5.34	84.24±3.83	83.62±7.64
ChNCs -PFTCS	87.21±3.78	84.75±1.16	82.08±2.84	79.04±5.12	86.12±9.58	88.71±0.81	89.41±0.52
ChNCs -PFTTS	73.37±1.48	71.55±5.46	60.59±4.53	44.69±4.00	36.04±0.45	33.06±2.02	37.52±2.63
ChNCs -PFDTS	99.00±5.00	90.77±5.73	100.41±3.86	96.95±3.15	94.47±0.78	95.31±1.81	97.35±3.49
ChNCs -DTS	150.47±6.33	128.87±7.84	101.22±0.34	111.61±4.29	104.79±18.43	101.72±6.52	83.68±6.15
s-SiO_2- ChNCs							
ChNCs 50 wt. %	148.01±1.65	146.07±7.74	147.43±2.97	144.79±2.43	134.21±1.36	132.57±5.20	132.13±5.90
ChNCs 10 wt. %	164.47±4.01	153.86±2.92	155.99±2.31	153.49±2.03	156.38±3.75	155.22±1.47	156.70±4.00
ChNCs 1.0 wt. %	162.67±1.53	151.16±1.77	156.76±1.62	154.17±2.32	152.56±0.59	154.61±1.92	150.61±4.13
ChNCs 0.1 wt. %	163.35±2.88	167.57±3.00	150.85±3.09	145.48±9.24	123.42±30.34	119.87±29.02	122.33±8.15
ChNCs 0 wt. %	162.83±2.10	159.84±1.09	149.29±4.15	145.05±12.76	110.79±15.68	118.16±25.52	101.03±12.64
s-TiO_2-ChNCs							
ChNCs 50 wt. %	155.08±3.66	154.22±8.52	156.81±5.32	138.41±7.85	121.94±10.41	124.80±23.64	131.29±16.00
ChNCs 10 wt. %	161.22±4.34	151.70±2.86	156.44±7.55	133.58±11.11	144.37±9.56	119.72±36.01	107.36±2.37
ChNCs 1.0 wt. %	156.12±2.25	158.75±3.11	153.41±6.79	143.92±17.77	139.78±12.05	139.53±10.82	92.05±5.20
ChNCs 0.1 wt. %	160.60±2.31	161.31±1.89	160.22±2.05	157.52±0.93	162.20±3.41	135.84±30.59	138.07±12.40
ChNCs 0 wt. %	162.48±5.25	158.66±2.59	154.50±6.69	151.06±1.33	148.88±12.34	129.46±8.56	126.75±17.20

Table S3. The contact angle (°) sliding angle (°) and contact angle hysteresis (°) after sea water immersion, immersion time range from 15 min to 120 min (n=3).

Time (min)	Contact Angle (°)	Sliding Angle (°)	Contact Angle Hysteresis (°)
0	158.22±3.86	0.37±0.06	1.13±0.06
15	157.31±3.12	0.43±0.06	1.53±0.15
30	156.65±3.05	0.57±0.06	1.80±0.60
45	152.29±1.57	0.53±4.76	1.80±0.30
60	153.86±2.11	0.67±1.32	3.40±1.06
75	153.68±2.26	0.93±1.16	3.93±0.96
90	151.76±2.15	1.27±5.46	4.33±1.31
105	151.48±2.25	1.67±5.73	6.40±0.95
120	150.55±2.70	2.17±7.84	8.67±1.08
